# Inhibition of Grain Growth by a Ce-rich Precipitate During the Annealing of Spray-Casted Magnesium Alloy

**DOI:** 10.3390/ma12050742

**Published:** 2019-03-04

**Authors:** Liang Liu, Huan Yu, Wei Yang, Zhitai Wang

**Affiliations:** National Defense Key Discipline Laboratory of Light Alloy Processing Science and Technology, Nanchang Hangkong University, Nanchang 330063, China; liuliangllly@163.com (L.L.); lmpfyh@163.com (H.Y.); wangzt@nchu.edu.cn (Z.W.)

**Keywords:** rapid solidification, microstructure refinement, precipitation, grain growth, magnesium alloy

## Abstract

The grain refinement and thermal stability behavior of a spray-casted AZ91D magnesium alloy were investigated with the addition of 0.75 wt% Ce. The results showed that the adoption of non-equilibrium solidification leads to remarkable grain refinement and the formation of a supersaturated solid solution, which suppresses the needle-like Al_11_Ce_3_ phase with a low cooling rate. After annealing at 420 °C, the grain morphology of rapidly solidified AZ91D+0.75Ce alloy changed from a granular to polygonal shape. Moreover, Mg_12_Ce particles precipitated and distributed homogeneously, which played an important role in grain boundary pinning at an elevated temperature. Consequently, the grain growth occurring during the isothermal annealing stage could be suppressed and the resultant grain size varied slightly, as did for the time, which increased from 2 h to 8 h.

## 1. Introduction

The applications of magnesium alloy present drastic growth with the need for power saving and environmental issues [1]. Amongst all the developed commercial alloys, AZ91D is one of the most promising materials with a noteworthy lightweight, good castability and an excellent electromagnetic shielding capacity [2,3,4]. According to the Hall–Petch relationship, the tensile strength of polycrystalline material has a pronounced dependence on the final grain size and often improves with the reduction of average grain size [5]. However, the magnesium alloy produced by a conventional ingot metallurgy method exhibits coarse grain due to the lack of effective inoculants, which thus generates an unsatisfactory strength and inferior corrosion resistance [6].

Non-equilibrium solidification has aroused continuous interest in the fields of materials science and condensed matter physics [7]. Due to the large departure from thermodynamic equilibrium, a remarkable grain refinement can be achieved by increasing the cooling rate during solidification without changing the initial alloy composition [8,9,10]. Meanwhile, solid solubility extension [11], meta-stable phase formation [12], as well as the elimination of detrimental intermetallic compounds [13] can be realized successfully, which lead to satisfactory microstructure modification. With the addition of a rare element, its mechanical property can be further improved due to solid solution hardening and precipitation hardening effect [14,15,16,17].

Nevertheless, the fine grain structure formed in non-equilibrium solidification suffers a great challenge due to the tendency of the grain growth to reduce their grain boundary energy, especially for the accelerated atomic diffusion rate at elevated temperature [18,19,20]. Up to now, it is commonly accepted that the thermal stability of fine grain microstructure can be improved by two methods, i.e., the exotic addition of insoluble inclusions [21] or the in situ formation of thermal stable precipitates [22]. In comparison with the former method, the precipitation of fine particles by controlling nucleation and growth processes seems more applicable because of the advantage of avoiding a non-uniform dispersion problem [23]. Using this method, Sheng et al. studied the mechanical properties of a rapidly solidified AZ91 alloy by forming thermally stable Mg_2_Si precipitates and an AlMg_2_Zn phase [24]. It was also reported that both the grain boundary sliding and dislocation motion can be inhibited effectively due to the presence of fine dispersed stable Al–La compound particles along the grain boundary [25].

While various methods have been reported for the grain refinement of magnesium alloy [26], investigations of the corresponding thermostability are still rather limited, especially for the relationship between the non-equilibrium effect and the subsequent solid state transformation [27]. In our previous study [18], it was reported that both the grain growth and the final grain size of a non-equilibrium solidified AZ91D alloy depended to a great extent on the isothermal time during the annealing process. The objective of present work is to investigate the microstructure evolution and thermostability behavior of an AZ91D+0.75Ce alloy by using copper mold spray-casting and isothermal annealing treatment. Special attention is paid to the non-equilibrium effect arising from a higher cooling rate during solidification and the subsequent precipitation, as well as the associated grain growth process.

## 2. Materials and Methods

The non-equilibrium solidification was performed by a copper mold spray-casting technique, which was conducted in an inert vacuum environment to prevent oxidation of the liquid metal. The commercial AZ91D magnesium alloy, coupled with 0.75 wt% Ce element, was selected in the present study due to its excellent grain refinement effect [23]. The mixture of raw materials was melted in a quartz crucible by a high-frequency induction heating apparatus and then injected into a copper mold with the inner and outer diameter of 8 mm and 80 mm, respectively. For the sake of comparison, the as-cast specimen was also prepared by in situ cooling in the crucible without injection. The isothermal annealing for the spray-casted alloy was performed in a heat-resistant furnace at 420 °C, under the protection of a flowing Ar atmosphere. To investigate the time dependence of the thermostability behavior of the spray-casted alloy, different holding periods (i.e., 2, 4, 6 and 8 h), were selected separately. The details of the experiment have been described elsewhere [18].

Microstructure analysis of the as-fabricated specimen was proceeded by optical microscopy (OM, VHX-600, KEYENCE, Osaka, Japan) after standard metallographic preparation techniques. The high magnification microstructure was observed by scanning electron microscope (SEM, Quantan 200, FEI, Oregon, America) and the detailed chemical composition was determined by the attached energy dispersive spectroscopy (EDS, INCA, Oxford Instruments, Oxford, UK) operating at 20 kV. To identify the morphology of tiny precipitate, a field emission scanning electron microscope (FE-SEM, Nova Nano 450, FEI, Oregon, America) with a point-to-point resolution of 1 nm was employed and the element distribution was characterized by energy dispersive spectroscopy (INCA 250 X-Max 50, Oxford Instruments, Oxford, UK), operating at 15 kV. To minimize the effect of electron–matter interaction volume from the matrix, the mode of Spot 2 was selected with the acquisition rate larger than 1.5 kcps and live time of 100 s. An X-ray diffractometer (XRD, D8 ADVANCE, Bruker-axs, Karlsruhe, Germany) was used to reveal the phase components in both the spray-casted alloy and that after being annealed for 2 h. Transmission electron microscopy (TEM, JEM 2100F, JEOL, Tokyo, Japan) operating at 200 kV was applied for high-resolution observation of the precipitate and the detailed phase structure was analyzed by the selected area electron diffraction method. The microhardness was measured by a Vickers indenter (DuraScan, Struers, Copenhagen, Denmark) with a load of 200 g and a dwell time of 15 s. Each of the samples was tested at five different areas and the average value was adopted in the present work.

## 3. Results and Discussion

### 3.1. Effect of the Cooling Rate on the Microstructure Evolution of the AZ91D+0.75Ce Alloy

**Figure 1:**
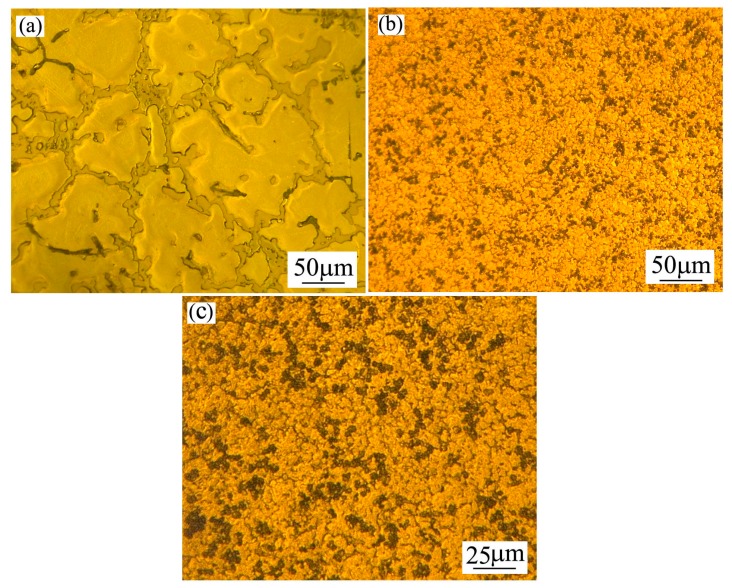
Optical microstructures of AZ91D+0.75Ce alloy fabricated by different methods: (**a**) As-cast; (**b**) spray-casting with low magnification and (**c**) spray-casting with high magnification.

Figure 1 shows the optical micrographs taken from the as-cast and spray-casted AZ91D+0.75Ce alloys, respectively. In the as-cast condition, coarse dendrites with a rosette-like morphology appeared, accompanied by the continuous distribution of the second phase along the grain boundary (Figure 1a). After measurement, the average grain size of the primary phase reached ~150 μm. In contrast, a remarkable grain refinement occurred in the spray-casted alloy (Figure 1b), and the fine granular with an average diameter of ~8 μm, could be observed from the enlarged structure (Figure 1c), which could be ascribed to the enhanced nucleation events and shortened grain growth period during the spray-casting process with a high cooling rate [28]. In addition, the accelerated migration rate for the liquid–solid interface favored microstructure stability and suppressed the formation of dendrite structure as well [29,30].

**Figure 2:**
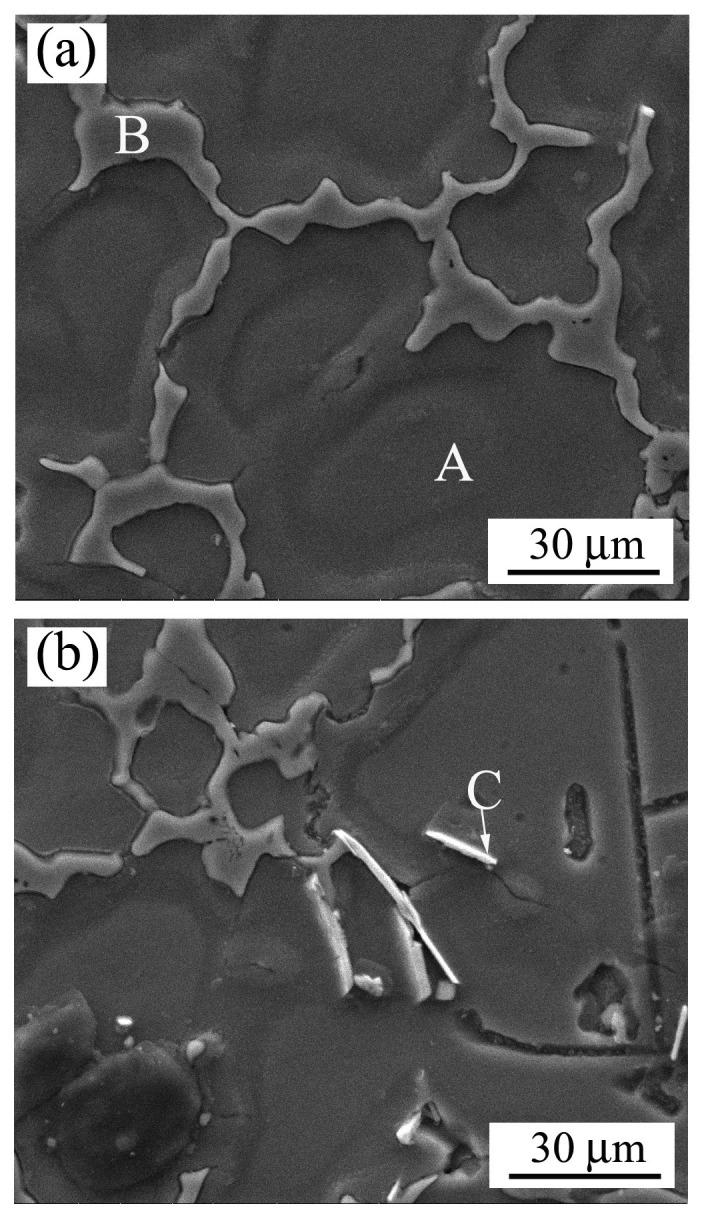
Scanning electron micrographs of as-cast AZ91D+0.75Ce alloy: (**a**) Matrix phase and grain boundary; (**b**) needle-like phase.

Figure 2 presents the SEM micrographs of the as-cast magnesium alloy, in which three different phases—labeled A, B, and C—are identified by EDS analysis and the results are listed in Table 1. After equipment calibration, it was verified that the measurement accuracy was nearly 90% for an element content larger than 1%, and more uncertainty existed for the minor alloying elements. As could be seen clearly, the main element in the matrix area was Mg (Point A in Figure 2a), thus the primary phase could be determined as α-Mg, according to the phase diagram [31]. However, a great amount of Al element existed in the grain boundary region (Point B in Figure 2a). According to the measured stoichiometry, it could be identified as the β phase, generating from serious solute segregation at the final solidification stage. As reported earlier [32], the brittle intermetallics were unfavorable to the mechanical strength, as well as the creep property at an elevated temperature. In addition, some needle-shaped phases could be observed in the high magnification microstructure, as indicated by Point C in Figure 2b. According to chemical analysis, it was revealed that both the Al and Ce elements were enriched with an atomic ratio of 4:1 and thus could be deduced as the Al_11_Ce_3_ phase, as reported in our previous work [32].

**Figure 3:**
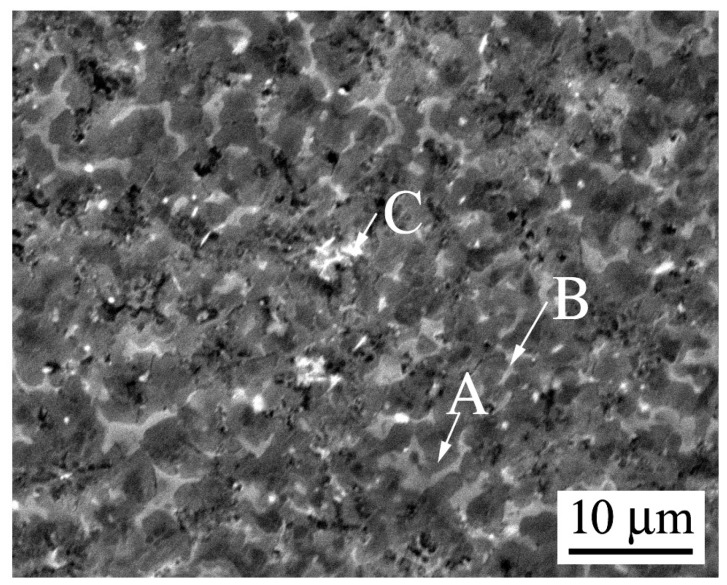
Scanning electron micrograph of spray-casted AZ91D+0.75Ce alloy.

After the copper mold spray-casting, both the solute distribution and the microstructure morphology of the AZ91D+0.75Ce alloy changed significantly, as evidenced by the SEM and EDS results shown in Figure 3 and Table 1. Firstly, the solute contents of Al, Ce and Mn in the *α*-Mg matrix increased due to the solute trapping effect (Point A) [33]. Secondly, the morphology of the *β* phase in the grain boundary appeared discontinuous (Point B). After measurement using Image-Pro Plus 6.0, its volume fraction herein was merely 7.53%, which was much less than that of 14.94% for the as-cast alloy. Generally speaking, the transition of grain morphology from coarse dendrite to fine granular favors the diffusion of solute atoms in the channel of residual liquid. Moreover, the formation of a supersaturation phase reduces the amount of solute atoms rejected from the as-solidified phase. As expected, the extent of solute segregation was weakened, which in turn resulted in the suppression of the *β* phase. Finally, both the length and volume fraction of the needle-like Al_11_Ce_3_ phase were decreased significantly, as verified by the occasional existence shown in Point C. It should be noted that both the Al content in *β* phase and Ce element in Al_11_Ce_3_ phase was much lower than that in Figure 2, which could be explained by the effect of the electron–matter interaction volume from the matrix and a larger uncertainty occurred for a smaller area herein. Regarding this fact, the influence of minor zinc as the solute was not considered in the present work, even though it has a strong impact on dendritic and eutectic phase formation [28].

### 3.2. Effect of Annealing on the Grain Growth and Precipitation of the Spray-Casted Alloy

**Figure 4:**
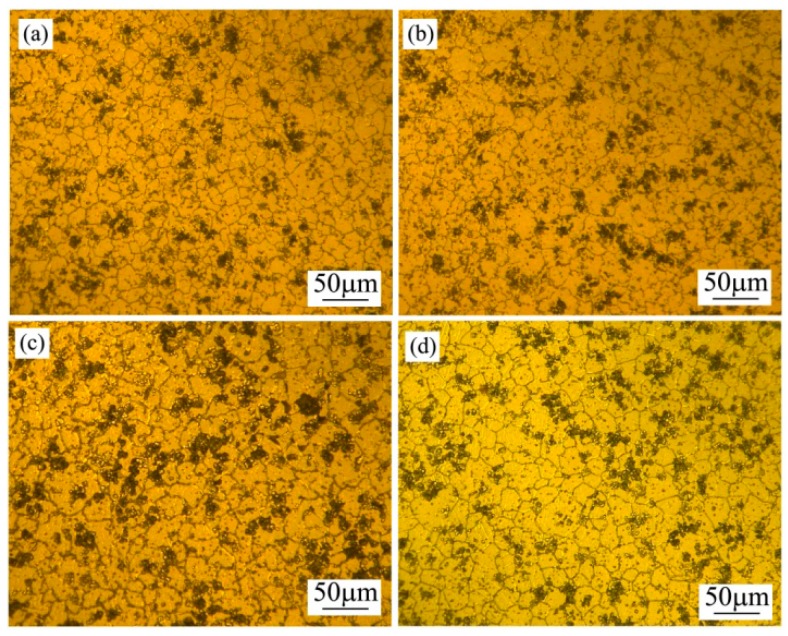
Optical microstructures of spray-casted AZ91D+0.75Ce alloy after being annealed at 420 °C for different times: (**a**) 2 h; (**b**) 4 h; (**c**) 6 h; (**d**) 8 h.

**Figure 5:**
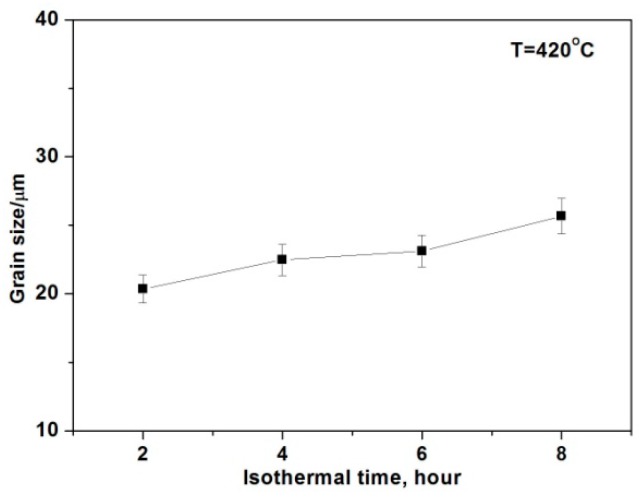
Average grain size of spray-casted AZ91D+0.75Ce alloy after annealed at 420 °C for different times.

Figure 4 presents the microstructure evolution of the spray-casted sample after being annealed at 420 °C for a different time. Even in the case of the shortest duration of 2 h, the granular morphology of the primary phase clearly changed to a polygonal shape and the *β* phase disappeared completely, due to its lower melting point temperature of merely 437 °C (Figure 4a). With increasing the annealing time, the size of the polygonal grain maintained constantly (Figure 4b–d) and the detailed variation is given in Figure 5 accordingly. As can be seen clearly, the average grain size was ~20 μm after being annealed for 2 h. With an extending the holding time, its value varied slightly and the final size reached ~26 μm, even for the longest time of 8 h, indicating a satisfactory thermostability behavior of the as-fabricated fine grain structure. As reported earlier, the average grain size of the spray-casted AZ91D alloy changed from ~35 μm to ~85 μm with increasing the holding time from 2 h to 8 h [18]. So, it could be deduced that the addition of Ce made the alloy less sensitive during exposure to an elevated temperature.

**Figure 6:**
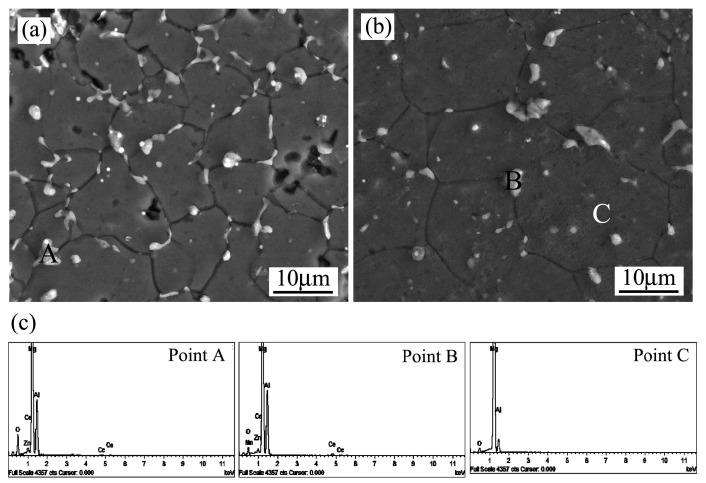
Field emission scanning electron micrographs of spray-casted AZ91D+0.75Ce alloy after annealed at 420 °C for different times: (**a**) 2 h; (**b**) 8 h; (**c**) EDS spectrums.

**Figure 7:**
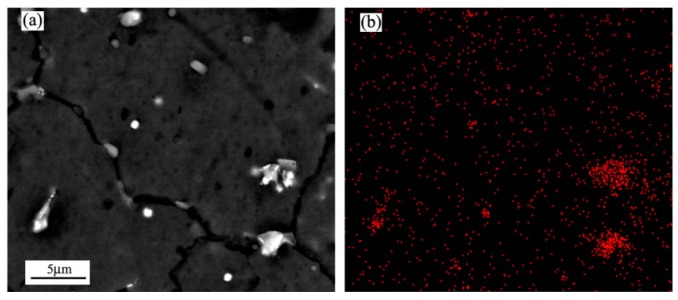
FE-SEM of the spray-casted AZ91D+0.75Ce alloy after being annealed at 420 °C for 8 h: (**a**) Field emission scanning electron micrograph; (**b**) the distribution of Ce element.

Although grain growth is inclined to occur upon annealing for spray-casted alloys [34], it can be suppressed effectively by the formation of tiny particles. In order to understand the underlying mechanism of the improved thermostability, Figure 6 shows the FE-SEM observations of the microstructure variation for the spray-casted alloy, after isothermal annealing for 2 h and 8 h, respectively. They were selected to represent the shortest and longest time for the studied annealing process. It is clearly shown that there were many fine blocks and they were well dispersed within the matrix, as well as at the corner of grain boundary (Figure 6a). According to the EDS measurements listed in Table 1, the amount of Mg element herein (Point A and Point B in Figure 6) was ~57 at%, which was much larger than the previous rare earth phase of 21.4 at% (Point C in Figure 2b). Moreover, the Al element reduced significantly, indicating a new phase rather than Al_11_Ce_3_ forms after annealing of spray casted alloy. rather than Al_11_Ce_3_, which formed after annealing of the spray-casted alloy. Additionally, the Mg content in the matrix increased as well (Point C in Figure 6a), which could be ascribed to the relief of microsegregation after the solid solution treatment. With extending the isothermal time, these Ce-rich particles still existed, except for a slight reduction of its volume fraction (Figure 6b). To verify the existence of Ce element in the particles, a mapping scanning for the specimen annealed for 8 h was performed on the FE-SEM observation, and the result is shown in Figure 7. As can be seen clearly, Ce was enriched for the bright phases embedded in the matrix and along the grain boundary, which indicated that these blocks with various sizes were indeed Ce-rich particles (Figure 7b).

**Figure 8:**
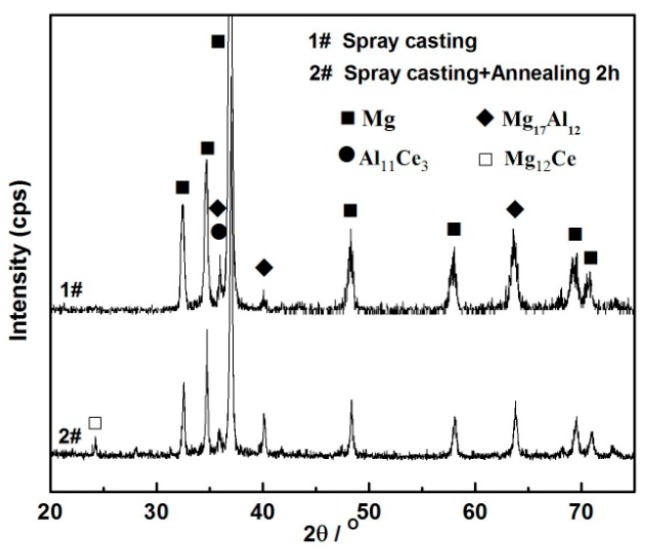
XRD patterns of spray-casted AZ91D+0.75Ce alloy and that after annealed at 420 °C for 2 h.

**Figure 9:**
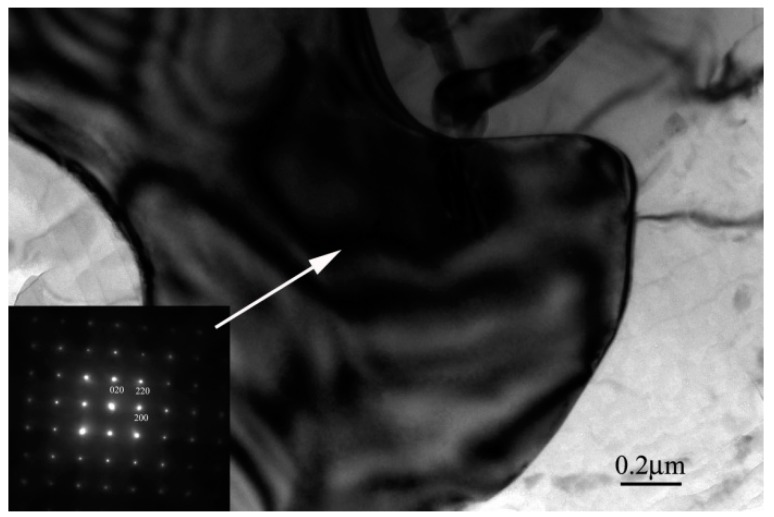
Transmission electron microscopy micrograph and selected area electron diffraction pattern of the formed Mg_12_Ce phase for the spray-casted AZ91D+0.75Ce alloy after being annealed at 420 °C for 2 h.

The structure of the Ce-rich compound was further analyzed by XRD, for the rapidly solidified AZ91D+0.75Ce alloy, after being annealed for 2 h, as shown in Figure 8. The main phase in the spray-casted alloy consisted of *α*-Mg, the *β* phase and a slight Al_11_Ce_3_ phase, which was in accordance with the observations shown in Figure 3. After the annealing treatment, a very limited amount of Mg_12_Ce could be identified, which was an indicator of the occurrence of precipitates. The phase was also reported in the single roller melt-spinning Mg_97_Ce_2_Zn_1_ alloy after being annealed at 400 °C [35]. This deduction was further supported by the result of a selected area electron diffraction, shown in Figure 9, where the particle at the grain boundary could be determined as the Mg_12_Ce phase with a tetragonal crystal structure.

Due to the supersaturation of the solute elements in the matrix being affected by the high cooling rate, the driving force for the nucleation of precipitates was enhanced accordingly. In addition, various defects, such as vacancies, dislocations and stacking faults, formed during the non-equilibrium solidification process and provided favorable sites for the initial nucleation in the subsequent precipitation process [5]. After comparison with the apparent grain growth behavior in the spray-casted AZ91D alloy without the Ce element [18], it could be inferred that the formation and presence of these Ce-rich particles served as obstacles to inhibit the grain growth under a high temperature, which led to the enhanced thermostability revealed in Figure 4. It should be noted that the particle size, spacing and morphology are all crucial factors for affecting the grain growth kinetics at elevated temperature [34]. So, proper consideration should be conducted in future work for describing the detailed pinning effect.

### 3.3. Micro-hardness of the As-prepared AZ91D+0.75Ce Alloys

**Figure 10:**
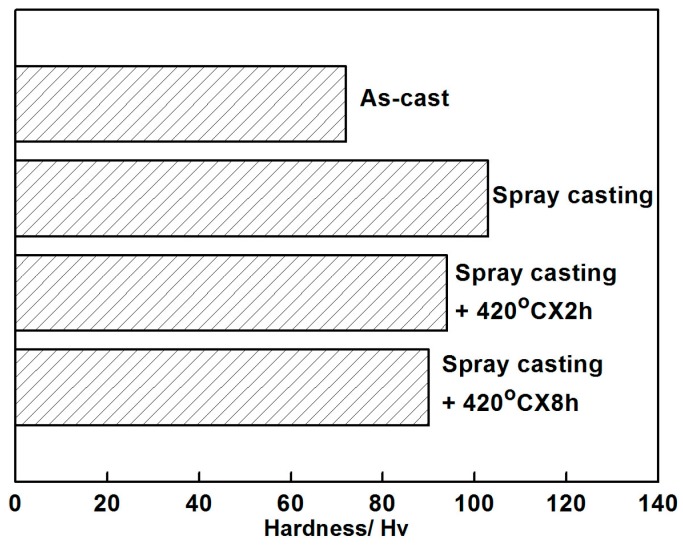
Microhardness of the AZ91D+0.75Ce alloys fabricated by different methods.

Figure 10 presents the microhardness of the as-prepared AZ91D+0.75Ce alloys with different methods. Due to the combined strengthening effects of the solid solution and grain refinement, a substantial enhancement of hardness was expected in the rapidly cooled alloy. It increased from 72 Hv for the as-casted alloy to 103 Hv for the spray-casted specimen. After an annealing treatment at 420 °C, a continuous decrease of hardness occurred, which could be ascribed to the dissolution of the *β* brittle phase and a slight grain growth when extending the holding time.

## 4. Conclusions

Copper mold spray-casting was adopted to investigate the microstructure evolution of an AZ91D+0.75Ce alloy. Subsequently, isothermal annealing was employed to indicate the thermal stability of the as-fabricated structure. The results obtained in this study are summarized as follows:

(1) The grain of the primary *α*-Mg in the spray-casted AZ91D+0.75Ce alloy was much finer than that in the as-cast condition. The non-equilibrium solidified microstructure presented a reduction of solute segregation and the suppression of the needle-like Al_11_Ce_3_ phase;
(2) After being annealed at 420 °C for 2 h, fine Ce-rich particles generated from the supersaturation solid solution and distributed homogeneously in the matrix, which could be inferred as the Mg_12_Ce phase. In addition, the grain morphology changed from a granular to polygonal shape, accompanied by the disappearance of the β phase at grain boundary;
(3) With increasing the annealing time to 8 h, these Ce-rich particles presented stability, which played an effective role in the inhibition of grain growth at an elevated temperature. As a consequence, the average grain size maintained between 20 μm and 26 μm after the annealing treatment, which indicated a satisfactory thermostability behavior of the fabricated fine grain structure;
(4) Both the solid solution strengthening and the fine grain strengthening improved the hardness of the spray-casted alloy with a high cooling rate. Moreover, its value decreased gradually after annealing at 420 °C, due to the dissolution of the *β* phase and weak grain growth.

## Figures and Tables

**Table 1:** EDS results for AZ91D+0.75Ce alloy fabricated by different methods (at percent).

Alloy	Point	Mg	Al	Zn	Ce	Mn	Si	O
As-cast	A	85.81	4.81	-	-	-	-	9.38
-	B	52.12	33.16	2.02	-	-	-	12.70
-	C	21.40	48.73	-	12.13	7.89	-	9.85
Spray-casting	A	73.49	12.25	-	0.71	0.82	-	12.73
-	B	72.60	17.28	-	0.76	0.75	-	8.61
-	C	79.94	9.21	-	1.65	-	-	9.20
Spray-casting + 420 ℃ × 2 h	A	57.79	23.97	0.62	1.14	-	0.65	15.83
-	B	57.41	29.50	0.78	1.07	0.29	-	10.95
-	C	85.48	8.00	-	-	-	-	6.52
-	-	-	-	-	-	-	-	-
